# Development and Validation of an Obstetric Comorbidity Risk Score for Clinical Use

**DOI:** 10.1089/whr.2021.0046

**Published:** 2021-11-02

**Authors:** Halley Ruppel, Vincent X. Liu, Patricia Kipnis, Monique M. Hedderson, Mara Greenberg, Heather Forquer, Brian Lawson, Gabriel J. Escobar

**Affiliations:** ^1^ Kaiser Permanente Northern California Division of Research, Oakland, California, USA.; ^2^ East Bay Department of Obstetrics and Gynecology, Kaiser Permanente Northern California, Oakland, California, USA.

**Keywords:** comorbidity summary score, maternal mortality, obstetrics, pregnancy complications, risk assessment, severe maternal morbidity

## Abstract

***Background:*** A comorbidity summary score may support early and systematic identification of women at high risk for adverse obstetric outcomes. The objective of this study was to conduct the initial development and validation of an obstetrics comorbidity risk score for automated implementation in the electronic health record (EHR) for clinical use.

***Methods:*** The score was developed and validated using EHR data for a retrospective cohort of pregnancies with delivery between 2010 and 2018 at Kaiser Permanente Northern California, an integrated health care system. The outcome used for model development consisted of adverse obstetric events from delivery hospitalization (*e.g.*, eclampsia, hemorrhage, death). Candidate predictors included maternal age, parity, multiple gestation, and any maternal diagnoses assigned in health care encounters in the 12 months before admission for delivery. We used penalized regression for variable selection, logistic regression to fit the model, and internal validation for model evaluation. We also evaluated prenatal model performance at 18 weeks of pregnancy.

***Results:*** The development cohort (*n* = 227,405 pregnancies) had an outcome rate of 3.8% and the validation cohort (*n* = 41,683) had an outcome rate of 2.9%. Of 276 candidate predictors, 37 were included in the final model. The final model had a validation c-statistic of 0.72 (95% confidence interval [CI] 0.70–0.73). When evaluated at 18 weeks of pregnancy, discrimination was modestly diminished (c-statistic 0.68 [95% CI 0.67–0.70]).

***Conclusions:*** The obstetric comorbidity score demonstrated good discrimination for adverse obstetric outcomes. After additional appropriate validation, the score can be automated in the EHR to support early identification of high-risk women and assist efforts to ensure risk-appropriate maternal care.

## Introduction

Rates of comorbidities among pregnant women are increasing and are associated with higher risk for adverse obstetric outcomes.^1–5^ Optimizing early identification of women with high-risk comorbidities may help address concerning trends in maternal outcomes in the United States.^6^ In prenatal care, risk assessment has historically focused on individual outcomes, such as risk for preeclampsia,^7,8^ and often relied on a handful of known comorbidity risk factors, such as diabetes or hypertension. As a result, current screening tools do not typically assess cumulative risk for adverse obstetric outcomes conferred by a woman's complete comorbidity profile.

One method for improving the systematic and early identification of high-risk women in clinical practice is by employing a composite comorbidity score.^9,10^ Commonly used comorbidity indices (*e.g.*, Charlson^11^ and Elixhauser^12^) were not developed for use in obstetrics and have limitations for use in this population. One comorbidity score created specifically for obstetrics, the Bateman Comorbidity Index, was developed for use in research from a Medicaid cohort of over 800,000 pregnancies.^13^ It was prospectively evaluated for clinical use during delivery hospitalization where it was manually calculated by the primary nurse.^10^

An obstetrics comorbidity score developed for automated calculation in the electronic health record (EHR) could help standardize allocation of existing health system services and resources, especially in the prenatal period, with no additional burden to clinicians. Using a specified risk threshold, women identified as high risk could be evaluated for additional prenatal monitoring services and for risk-appropriate level of maternal care.^14^ Such a score would likely be most useful in the outpatient setting. In the current study, we sought to develop and validate an Obstetrics Comorbidity Score that can be implemented in the EHR for real-time calculation.

We conducted the study in Kaiser Permanente Northern California (KPNC), an integrated health care system with around 40,000 deliveries each year. Automated risk scores are widely used in other contexts within KPNC both for risk adjustment and to identify patient risk in real-time.^15–18^

## Materials and Methods

### Study design and population

The KPNC Institutional Review Board for the Protection of Human Subjects determined the study exempt. We used a retrospective cohort design. We defined the cohort for this study using an existing dataset of deliveries^19^ from 15 KPNC hospitals between 2010 and 2018 that was originally created for development of KPNC's real-time inpatient obstetric early warning system, which is now undergoing piloting.^20^ The dataset included deliveries in which gestational age at admission was ≥22 weeks and excluded deliveries where fetal demise occurred before admission. The dataset is described in depth elsewhere.^19^

### Outcome

To develop the Obstetric Comorbidity Score, we used a composite binary outcome for the occurrence of an adverse maternal or neonatal event during delivery hospitalization. The composite outcome included adverse maternal and neonatal events that were previously defined for the existing dataset by clinician experts using a combination of structured EHR data, chart review, and administrative codes.^19^ Compared with studies that rely on administrative data alone, the availability of EHR data allowed for: (1) more detailed definition of adverse outcomes associated with delivery; (2) comprehensive capture of both adverse maternal and neonatal outcomes; and (3) reduced risk of misclassification.

The adverse maternal and neonatal events comprising the composite outcome included: severe preeclampsia, severe preeclampsia with major deterioration, eclampsia, severe hemorrhage, embolism, transfer to intensive care, major deterioration, uterine rupture, fetal death, hypoxic–ischemic encephalopathy, neonatal acidosis, and maternal death (within 42 days of delivery). Definitions are reported elsewhere.^19^ The distribution of the adverse events in our dataset are in Supplementary Table S1.

### Candidate predictors

For each delivery in the existing dataset, we obtained diagnoses documented during all health care encounters in the 12-month period before, but excluding, delivery hospitalization. We used International Classification of Diseases (ICD) diagnosis codes as entered, recognizing that there may be heterogeneity in coding practices. We grouped diagnoses using the Healthcare Cost and Utilization Project (HCUP) Clinical Classification Software (CCS) for ICD-9-CM and ICD-10-CM beta version.^21,22^

We chose to use HCUP CCS groups as candidate predictors for our model because the purpose of the study was to develop a comorbidity score that could be readily implemented in the EHR. HCUP CCS groups are already used in KPNC's EHR system (Epic, Verona, WI), reducing barriers to implementation related to manually coding individual ICD codes. Epic is also one of the most widely used EHR systems in the United States. Of the 284 CCS groups, 272 occurred in our dataset. More than 15 of the CCS groups are directly related to conditions of pregnancy. We also included four additional candidate predictors important to obstetric outcomes: multiple gestation, obesity, maternal age, and parity (see Supplementary Table S2 for definitions).

### Statistical analysis

We developed the Obstetric Comorbidity Score using data from pregnancies in which the delivery occurred between January 1, 2010 and March 31, 2017. We retained the last year of deliveries, April 1, 2017–March 31, 2018, for validation. To ensure complete data for model development, we restricted inclusion to those who had been a member of KPNC for ≥10 months before delivery. However, we did not use this restriction in the validation cohort, to reflect how the score would be used prospectively.

Because of the large number of CCS groups, many of which are not typically relevant to pregnant women (*e.g.*, dementia), we used least absolute shrinkage and selection operator (LASSO; L1 penalized logistic regression) to identify the key variables associated with increased risk for the outcome. Penalized regression removes predictors from the model by shrinking coefficients to 0 for the least important candidate predictors.^23^ To select the optimal *λ* tuning parameter (using 1-standard error threshold), we used 10-fold crossvalidation.^24^

We then fit a second model (logistic regression) using the variables selected by LASSO that were associated with an increased risk for the outcome (including all levels of categorical variables), which did not substantially change model performance. We estimated out-of-sample error in logistic regression using 10-fold cross-validation. We then evaluated performance of the final Obstetric Comorbidity Score model in the validation cohort.

We employed several additional approaches to evaluate our model in the validation cohort. We compared the performance of the Obstetric Comorbidity Score with that of two other methods of identifying high-risk women (the Bateman Comorbidity Index^13^ and a “simple risk factor” approach). The Bateman Comorbidity Index is an obstetric-specific comorbidity index widely used in obstetrics research.^13^ Scores are assigned based on 21 weighted variables (comorbidities/conditions and maternal age) and can range from 0 to 45. We calculated Bateman scores for each pregnancy in our cohort and evaluated capture of our outcome at two different threshold cutoffs (≥6 and, less restrictively, ≥2). In a recent study, a Bateman score of ≥6 was used as the threshold cutoff for alerting the provider.^10^ We also compared the Obstetric Comorbidity Score with a “simple risk factor” approach by flagging women as high risk if they had any of four well-known individual risk factors: diabetes, hypertension, advanced maternal age (≥40 years), or multiple gestation.

We also assessed performance of the Obstetric Comorbidity Score and the simple risk factor approach at 18 weeks of pregnancy, because early identification of high-risk women would allow better prenatal monitoring and delivery planning. To do this, we obtained predictor data from health care encounters during the 12 months before 18 weeks gestation for each pregnancy and used the Obstetric Comorbidity Score to generate predicted probabilities.

We conducted all analyses using R version 4.0.0. We assessed model performance based on discrimination (area under the receiver-operator characteristic curve [c-statistic or AUC]), calibration (by examining plots of the actual outcome rate compared with the predicted probabilities by decile), and Brier scores for model accuracy (the mean of the squared differences between the predicted probability and actual outcome).^25^ We generated AUCs for the simple known risk factors (as a composite binary predictor) and the Bateman Comorbidity Index using logistic regression, with our composite outcome as the dependent variable.

Our goal is to use the model to assign women to either high- or low-risk groups and we therefore calculated sensitivity, specificity, positive predictive value (PPV), and number needed to evaluate (1/PPV, NNE) for comparison at several risk threshold cutoffs. NNE, also called number needed to screen,^26^ represents the amount of work required by the clinician and health system to find each true case.^27^

For the purposes of initial evaluation of the tool, we also compared the Obstetric Comorbidity Score with the Bateman Comorbidity Index and the “simple risk factor” approach using the Obstetric Comorbidity Score threshold where sensitivity approximately matched the sensitivity of the other score. This allowed us to determine whether, for the same sensitivity, the Obstetric Comorbidity Score was more efficient in terms of specificity, PPV, and NNE. The purpose of this analysis was to explore whether our data-driven approach, which incorporated more variables than either the Bateman Index or the “simple risk factor” approach, would be more efficient in terms of specificity, PPV, and NNE than the other methods. However, this analysis was intended for initial evaluation purposes only and was not intended to be used to select thresholds for use in clinical practice. Further evaluation and clinical judgment will be needed by clinical sites to determine the threshold with the sensitivity and specificity that would be both safe and effective for clinical use.

## Results

The development cohort included 227,405 pregnancies. The mean maternal age was 31.2 (standard deviation ±5.6) years, 40.8% of pregnancies were among women who were white, 24.9% Asian, 24.1% Hispanic, and 7.3% Black (Table 1). About one-quarter (26.7%) delivered by cesarean section. In the validation cohort (*n* = 41,683 pregnancies) demographics were largely similar. The composite outcome occurred in 3.8% (8,726) of pregnancies in the development cohort, compared with 2.9% (1,201) of pregnancies in the validation cohort. The lower outcome rate in the validation cohort was primarily driven by a lower rate of severe preeclampsia (Supplementary Table S1). We did not restrict KPNC membership duration in the validation cohort and 83.8% (*n* = 34,938) were KPNC members for ≥10 months before admission for delivery.

**Table 1.** Pregnancy Characteristics in Development and Validation Cohorts^a^

Characteristic	Development cohort (*n* = 227,405)	Validation cohort (*n* = 41,683)
Maternal age, years Mean ± SD Median [IQR]	31.2 ± 5.6 31.0 [28.0, 35.0]	31.6 ± 5.3 32.0 [28.0, 35.0]
Advanced maternal age (≥40 years)	13,881 (6.1)	2,539 (6.1)
Race/ethnicity		
American Indian/Eskimo	749 (0.3)	157 (0.4)
Asian/Pacific Islander (non-Hawaii)	56,637 (24.9)	10,726 (25.7)
Black	16,657 (7.3)	3,068 (7.4)
Hispanic	54,887 (24.1)	10,340 (24.8)
Pacific Islander (Hawaii)	2,236 (1.0)	496 (1.2)
White	92,683 (40.8)	15,986 (38.4)
Other/unknown	3,556 (1.6)	910 (2.2)
Delivery through cesarean section	60,642 (26.7)	11,043 (26.5)
Gestational age at admission for delivery hospitalization, weeks Mean ± SD Median [IQR]	39.2 ± 1.9 39.4 [38.6, 40.3]	39.1 ± 1.9 39.4 [38.6, 40.3]
Parity^b^ (median [IQR])	1 [0,1]	1 [0,1]
Bateman Comorbidity Index score Mean ± SD Median [IQR]	0.9 ± 1.2 0 [0,1]	0.9 ± 1.2 1 [0,1]
Composite outcome (adverse obstetric event)^c^	8,726 (3.8)	1,201 (2.9)

^a^ Table values are mean ± SD and median [IQR] for maternal age, gestational age, Bateman Comorbidity Index score; median [IQR] for parity; and *n* (column %) for categorical variables; may not sum to 100% due to rounding.

^b^ Parity was missing in 74 pregnancies and was imputed to median (1).

^c^ Components of composite outcome can be found in Supplementary Table S1.

IQR, interquartile range; SD, standard deviation.

### Obstetric Comorbidity Score development

In total, 272 of the 284 CCS groups were present in the development cohort. In addition to the 272 CCS predictors, we included parity, multiple gestation, obesity, and maternal age as candidate predictors in the LASSO model. Of these 276 candidate predictors, 37 were included in the final model. Table 2 displays the distribution of the top 10 most frequently occurring predictors from the final Obstetric Comorbidity Score model in the development and validation cohorts and their estimated effects. Several predictors had notably higher rates in the validation than development cohort (*e.g.*, multiple gestation, obesity, CCS181 “other complications of pregnancy”) (see Supplementary Table S3 for full list of model predictors).

**Table 2.** Top 10 Most Frequently Occurring Obstetric Comorbidity Score Predictors^a^

Predictor^b^	Development^c^ *n* = 227,405	Validation^c^ *n* = 41,683	Beta coefficient	Adjusted odds ratio	95% CI
CCS 195: Other complications of birth	129,335 (56.9)	25,139 (60.3)	0.20	1.22	1.16–1.29
CCS 181: Other complications of pregnancy	125,426 (55.2)	28,682 (68.8)	0.05	1.05	1.00–1.10
Parity^d^					
0	101,015 (44.4)	18,587 (44.6)	0.91	2.48	2.35–2.62
1–4	124,624 (54.8)	22,740 (54.6)	Reference		
≥5	1,766 (0.8)	356 (0.9)	0.17	1.19	0.93–1.52
Maternal age, years					
≤19	5,581 (2.5)	510 (1.2)	0.24	1.27	1.10–1.46
20–24	22,856 (10.1)	3,749 (9.0)	0.20	1.22	1.12–1.33
25–29	52,650 (23.2)	9,569 (23.0)	Reference		
30–34	81,972 (36.0)	15,148 (36.3)	0.05	1.05	0.98–1.12
35–39	50,465 (22.2)	10,168 (24.4)	0.21	1.24	1.15–1.33
40–44	12,820 (5.6)	2,366 (5.7)	0.44	1.55	1.41–1.71
≥45	1,061 (0.5)	173 (0.4)	0.80	2.22	1.77–2.79
CCS 182: Hemorrhage during pregnancy; abruption placenta; placenta previa	41,836 (18.4)	6,872 (16.5)	0.07	1.08	1.02–1.14
CCS 159: Urinary tract infections	34,213 (15.0)	6,676 (16.0)	0.07	1.07	1.01–1.14
CCS 189: Previous cesarean section	29,899 (13.1)	5,233 (12.6)	0.21	1.24	1.14–1.34
CCS 186: Diabetes or abnormal glucose tolerance complicating pregnancy	29,376 (12.9)	5,855 (14.0)	0.22	1.25	1.17–1.33
CCS 251: Abdominal pain	27,048 (11.9)	4,781 (11.5)	0.06	1.06	0.99–1.13
CCS 163: Genitourinary symptoms and ill-defined conditions	26,942 (11.8)	5,321 (12.8)	0.07	1.08	1.01–1.15

^a^ Full list of Obstetric Comorbidity Score predictors can be found in Supplementary Table S3.

^b^ CCS descriptions have been abbreviated.

^c^ Table values are *n* (column %); may not sum to 100% due to rounding (parity, maternal age).

^d^ Parity was missing in 74 observations; imputed to median (1).

CCS, Healthcare Cost and Utilization Project Clinical Classification Software group.

### Obstetric Comorbidity Score performance

**FIG. 1.**
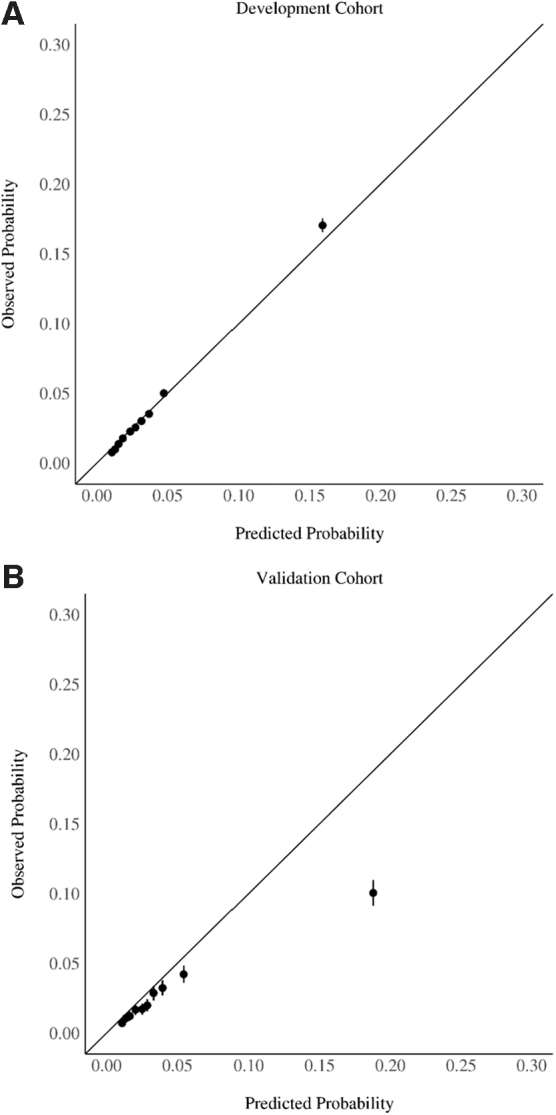
Observed (actual) adverse obstetric outcome rate compared with the predicted probability of an adverse obstetric outcome, by decile, in the development cohort (*n* = 227,405) **(A)** and the validation cohort (*n* = 41,683) **(B)**.

The Obstetric Comorbidity Score demonstrated good discrimination, with a cross-validated AUC of 0.76 (95% confidence interval [CI] 0.76–0.77) in the development cohort and an AUC of 0.72 (95% CI 0.70–0.73) in the validation cohort. The model was well calibrated in the development cohort, although it exhibited more overestimation of risk in the validation cohort at higher predicted probabilities (Fig. 1). The Brier score was 0.03 in both cohorts.

At the same risk thresholds, sensitivity and specificity were slightly lower in the validation cohort compared with the development cohort; for example, using 5% risk cutoff, sensitivity in the development cohort was 49.6%, and specificity was 88.3%, compared with sensitivity of 45.8% and specificity of 84.3% in the validation cohort (Table 3). Table 3 displays the sensitivity, specificity, PPV, and NNE of the Obstetric Comorbidity Score at several example risk thresholds. In the Supplementary Figure S1, we present the sensitivity, specificity, and PPV of the Obstetric Comorbidity Score across a range of risk thresholds in the development and validation cohorts.

**Table 3.** Performance of Obstetrics Comorbidity Score in Development and Validation Cohorts

Cohort	Outcome rate (%)	AUC (95% CI)	Brier score	Risk threshold^a^	Sensitivity (%)	Specificity (%)	PPV (%)	NNE	% pregnancies crossing threshold
**Development**	3.8	0.76 (0.76–0.77)	0.03	3%	73.9	62.2	7.2	14	39.2
				4%	58.0	81.1	10.9	9	20.4
				5%	49.6	88.3	14.5	7	13.2
**Validation**	2.9	0.72 (0.70–0.73)	0.03	3%	72.1	58.2	4.9	21	42.7
				4%	54.8	76.4	6.5	16	24.5
				5%	45.8	84.3	8.0	13	16.6

^a^ Risk threshold used to calculate sensitivity, specificity, PPV, NNE, and % of pregnancies crossing threshold.

AUC, area under the receiver-operator characteristic curve; CI, confidence interval; NNE, number needed to evaluate (1/PPV); PPV, positive predictive value.

### Comparison of Obstetric Comorbidity Score to Bateman Comorbidity Index

In our validation cohort, the Bateman Comorbidity Index scores had an AUC of 0.63 (95% CI 0.62–0.65) for our outcome (“A” in Table 4). Using a Bateman score cutoff of ≥6 resulted in a sensitivity of just 4.25% for our outcome, whereas a cutoff of ≥2 yielded a sensitivity of 44.5%. At a similar sensitivity (44.3%), the Obstetric Comorbidity Score had higher specificity (84.9%) and PPV (8.0%) than the Bateman Comorbidity Index (specificity = 76.1%; PPV = 5.2%).

**Table 4.** Comparison of Obstetric Comorbidity Score to Bateman Comorbidity Index and Simple Risk Factors in Validation Cohort

Model	AUC (95% CI)	Sensitivity (%)	Specificity (%)	PPV (%)	NNE	% pregnancies crossing threshold
*A. Comparison to Bateman Comorbidity Index*						
Obstetric Comorbidity Score^a^ (5.1% risk threshold)	0.72 (0.70–0.73)	44.3	84.9	8.0	13	15.9
Bateman Comorbidity Index, cutoff score ≥2	0.63 (0.62–0.65)	44.5	76.1	5.2	19	24.5
*B. Comparison to simple risk factors*						
Admission for delivery^b^						
Obstetric Comorbidity Score^c^ (5.5% risk threshold)	0.72 (0.70–0.73)	41.5	86.6	8.4	12	14.2
Simple risk factors^d^	0.57 (0.55–0.58)	41.5	72.2	4.2	24	28.2
18 weeks gestation^b^						
Obstetric Comorbidity Score^c^ (4.5% risk threshold)	0.68 (0.67–0.70)	28.5	89.9	7.7	13	10.6
Simple risk factors^d^	0.57 (0.55–0.58)	28.6	84.9	5.3	19	15.5

^a^ Selected threshold cutoff that had a sensitivity similar to the sensitivity of the Bateman Comorbidity Index using cutoff score ≥2.

^b^ Values calculated using predictor data identified from all health care encounters in the 12-month period before respective time point.

^c^ Selected threshold cutoff that had a sensitivity similar to the sensitivity of the simple risk factors.

^d^ Hypertension (CCS 98 or CCS 99) and/or diabetes (CCS 49 or CCS 50 or CCS 186) and/or multiple gestation (see Supplementary Table S2) and/or maternal age ≥40.

### Comparison to simple risk factors at admission for delivery and 18 weeks of pregnancy

In the validation cohort, the AUC of the simple risk factors (diabetes and/or hypertension and/or advanced maternal age and/or multiple gestation) was 0.57 (95% CI 0.55–0.58) (“B” in Table 4). The sensitivity of the simple risk factors was 41.5% and at the same sensitivity, the Obstetric Comorbidity Score demonstrated a higher specificity (86.6% vs. 72.2%) and PPV (8.4% vs. 4.2%).

Although we developed the Obstetric Comorbidity Score using predictor data from health care encounters during the 12-month period before admission for delivery, we also assessed Obstetric Comorbidity Score performance using predictor data from health care encounters during the 12 months before 18 weeks of pregnancy. We found a modest decrease in Obstetric Comorbidity Score discrimination (AUC = 0.68, 95% CI 0.67–0.70) and no change in the simple risk factor discrimination (AUC 0.57) at 18 weeks of pregnancy (“B” in Table 4). The known risk factor approach identified 28.6% of outcomes and at approximately the same sensitivity (28.5%), the Obstetric Comorbidity Score had higher specificity (89.9% vs. 84.9%) and PPV (7.7% vs. 5.3%).

In Supplementary Table S4, we exhibit the suboutcomes identified by the Obstetric Comorbidity Score, the Bateman Comorbidity Index, and the simple risk factors. We found that at a similar sensitivity for the composite outcome, the Obstetric Comorbidity Score identified fewer of the outcomes that were not preeclampsia or eclampsia.

## Discussion

Using a data-driven approach, we developed an obstetrics comorbidity risk score from a cohort of 227,405 pregnancies. We designed the score to be integrated seamlessly within an EHR by using existing diagnosis groupings (HCUP CCS). The Obstetric Comorbidity Score demonstrated good discrimination in both the development cohort (AUC 0.76) and a temporally distinct validation cohort (AUC 0.72), and good calibration in the range where clinical decisions are most likely to be made. For our outcome, the Obstetric Comorbidity Score was more efficient at a similar sensitivity than the existing Bateman Comorbidity Index or a simple approach of identifying high-risk women based on individual well-known risk factors.

The Obstetric Comorbidity Score can be used to stratify high-risk pregnancies at admission for delivery or earlier in pregnancy. The score can help support efforts to provide risk-appropriate levels of maternal care, as recommended in the *Levels of Maternal Care Obstetric Care Consensus,* and address growing concern that increasing rates of comorbid disease are impacting maternal outcomes.^14^

Many risk scores exist for clinical use in obstetrics, but they are typically focused on deterioration during delivery hospitalization^19,28–31^ or are condition-specific scores for use in the prenatal period, such as those designed to predict pre-eclampsia or gestational diabetes.^7,8,32^ We created a comprehensive Obstetric Comorbidity Score that offers global, versus condition-specific, risk assessment. The goal of the score is to support health system-level triage to appropriate maternal care resources, particularly in the prenatal period. However, the Obstetric Comorbidity Score could also be used as one component of an in-patient early warning score that also incorporates EHR data such as vital signs and laboratory values.^17,19^

Our work complements and extends prior work developing obstetric comorbidity risk scores, which has primarily focused on risk adjustment for research.^13,33^ The most widely used risk adjustment score, the Bateman Comorbidity Index, demonstrates consistent performance in validation in retrospective datasets (AUC 0.64–0.66).^9,13,34^ A manually calculated, modified Bateman Comorbidity Index was also prospectively evaluated during delivery hospitalization in 2,828 women and reported to have an AUC of 0.83.^10^ Leonard et al.^33^ recently used machine learning to develop an obstetrics comorbidity score for risk adjustment, demonstrating an AUC of 0.84 in a dataset from California.

Both existing risk scores used a hand-curated set of risk factors selected from ICD codes, and Leonard et al.^33^ used diagnoses only from the delivery hospitalization itself. In contrast, we used a data-driven approach to establish risk factors from existing diagnosis groupings (HCUP CCS) and identified risk factors from all encounters in the year before admission. Together with existing studies, our work demonstrates a proof of concept—that developing a comorbidity score from ICD data is possible and predictive of poor outcomes and may have clinical utility.

To assess whether our approach had added benefit in our population over existing comorbidity scores, we evaluated the Bateman Comorbidity Index in our cohort. Although we defined our outcome differently than that used to develop the original Bateman Comorbidity Index, we found that the Bateman Comorbidity Index demonstrated a similar AUC in our cohort to prior validations (AUC 0.63).^9,34^ Our Obstetric Comorbidity Score demonstrated a higher AUC (0.72); however, it is important to note that at a similar sensitivity, the Bateman Comorbidity Index captured more of the outcomes in our cohort that were not preeclampsia/eclampsia than did the Obstetric Comorbidity Score. We did not have the resources to evaluate performance of Leonard et al's^33^ comorbidity score in our cohort.

### Clinical implications

Many adverse maternal outcomes are preventable and early identification of high-risk women is key.^35,36^ The primary clinical utility of the Obstetric Comorbidity Score is likely in the prenatal period. The score can be used to standardize early identification of higher-risk women who should be evaluated for existing services within the health care system, such as more intensive prenatal monitoring, provider type, virtual versus in-person prenatal visits, and delivery location.

The Obstetric Comorbidity Score is intended to be used to support population-wide identification of high-risk women and prompt further evaluation, not to replace clinician judgment.

The Obstetric Comorbidity Score may be especially helpful for clinicians who typically work with low-risk women, to ensure those who need more intensive prenatal care are not missed. Before clinical use, further validation and evaluation are needed. In particular, a risk threshold to define “high risk” must be selected that is appropriate for the health system context (*e.g.*, resources, costs, and implications of false positives^26^), taking into account the sensitivity/specificity tradeoff.

### Research implications

In the current study, we developed and internally validated the Obstetric Comorbidity Score. We also compared performance of the Obstetric Comorbidity Score with the Bateman Comorbidity Index and a “simple risk factor” approach, by matching the sensitivity and comparing specificity, PPV, and NNE. Although this analysis was useful in the context of this initial validation study, the risk thresholds presented are not intended for clinical use as they were not optimized for patient care. If inappropriate risk thresholds are implemented into patient care, detectable true cases could be missed. Rigorous evaluation is needed to determine risk thresholds that would be appropriate for clinical care.

Before clinical implementation, the Obstetric Comorbidity Score should be externally validated. Additionally, we developed the Obstetric Comorbidity Score using a retrospective cohort, and prospective evaluation of the clinical impact of using the Obstetric Comorbidity Score is also needed. Due to the potential for bias in any predictive model developed using existing datasets, evaluation of the Obstetric Comorbidity Score across racial/ethnic groups is critical before implementation and on an ongoing basis. Following additional validation, health care systems would need to engage in the work of determining a clinically appropriate risk thresholds for flagging “high-risk” women based on sensitivity/specificity trade-off, the unique features of their clinical practice, and the patient population they serve.

### Strengths and limitations

Strengths of our study include use of HCUP CCS groups as predictors to facilitate OCS implementation in the EHR. HCUP CCS groups already exist in Epic, which is one of the most commonly used EHR systems. However, the use of an existing classification structure also has limitations, due to the variety of diagnoses captured in each HCUP CCS group. We developed the score using predictor data that were documented (thus known) before delivery hospitalization. Our data-driven approach may have elucidated less obvious risk factors, although it is subject to the treatment paradox.^37^ Using a more contemporary validation cohort than development cohort allowed us to approximate future performance of the Obstetric Comorbidity Score. Although the rate of the outcome and some predictors was markedly different between the development and validation cohort, the Obstetric Comorbidity Score maintained good discrimination. Some of the variation in predictor frequency across the cohorts may be due to temporal changes in the pregnant population and care, underscoring the importance of ongoing monitoring of Obstetric Comorbidity Score performance if used in real-time.

The Obstetric Comorbidity Score, like other existing comorbidity scores, relies on diagnosis codes, with inherent limitations, including misclassification. Additionally, we developed the Obstetric Comorbidity Score in an insured population in an integrated health care system. For institutions without comprehensive EHRs or access to maternal records from which to automate the score, a manually calculable score would be more appropriate.

## Conclusion

We developed a comorbidity score for obstetrics for implementation in the EHR that was more efficient at predicting adverse outcomes in our population compared with other currently available methods of risk assignment. External and prospective evaluations of the Obstetric Comorbidity Score are warranted. Early and systematic identification of high-risk women will support efforts to provide risk-appropriate levels of maternal care and help address the national crisis in maternal morbidity and mortality.

## Supplementary Material

Supplemental data

Supplemental data

Supplemental data

Supplemental data

Supplemental data

## Abbreviations Used

AUC: area under the receiver-operator characteristic curve
BIRCWH: Building Interdisciplinary Research Careers in Women's Health
CCS: Clinical Classification Software
CI: confidence interval
EHR: electronic health record
HCUP: Healthcare Cost and Utilization Project
IQR: interquartile range
KPNC: Kaiser Permanente Northern California
LASSO: least absolute shrinkage and selection operator
NNE: number needed to evaluate (1/PPV)
PPV: positive predictive value
SD: standard deviation
