# Supplementary material for: Development and Validation of an Obstetric Comorbidity Risk Score for Clinical Use
Source: Womens Health Rep (New Rochelle). 2021 Nov 2;2(1):507–15. doi: 10.1089/whr.2021.0046 (PMC8617587; doi:10.1089/whr.2021.0046)
Supplement: Supplemental data [file Suppl_TableS1.docx]

**Supplemental Table 1. Distribution of outcome, and its elements, in development and validation cohorts ^a^**

| **Outcome ^b^** | **Development cohort**  **(n= 227,405)** | **Validation cohort**  **(n=41,683)** |
| --- | --- | --- |
| **Composite outcome ^c^** | 8726 (3.84) | 1201 (2.88) |
| Pre-eclampsia with severe features, antepartum | 6357 (2.80) | 651 (1.56) |
| Pre-eclampsia with severe features, postpartum | 488 (0.21) | 49 (0.12) |
| Eclampsia, antepartum | 11 (0.00) | 2 (0.00) |
| Eclampsia, postpartum | 5 (0.00) | 0 (0.00) |
| Fetal death | 53 (0.02) | 26 (0.06) |
| Neonatal acidosis | 782 (0.34) | 188 (0.45) |
| Neonatal hypoxic-ischemic encephalopathy | 277 (0.12) | 67 (0.16) |
| Embolism, antepartum | 11 (0.00) | 4 (0.01) |
| Embolism, postpartum | 21 (0.01) | 2 (0.00) |
| Severe hemorrhage, antepartum | 87 (0.04) | 17 (0.04) |
| Severe hemorrhage, postpartum | 721 (0.32) | 146 (0.35) |
| Intensive care unit transfer, antepartum | 31 (0.01) | 8 (0.02) |
| Intensive care unit transfer, postpartum | 443 (0.19) | 128 (0.31) |
| Major deterioration without intensive care unit transfer, antepartum | 21 (0.01) | 17 (0.04) |
| Major deterioration without intensive care unit transfer, postpartum | 213 (0.09) | 69 (0.17) |
| Maternal death | 15 (0.01) | 5 (0.01) |
| Uterine rupture | 219 (0.10) | 51 (0.12) |

**^a^** Table values are n (%)

**^b^** Outcome definitions can be found in Escobar et al,^2^ except for pre-eclampsia with severe features, which was defined for the current study as systolic or diastolic blood pressure criteria for pre-eclampsia and elevated relevant laboratory results.

**^c^** Sub-outcomes are not mutually exclusive
