# Supplementary material for: Development and Validation of an Obstetric Comorbidity Risk Score for Clinical Use
Source: Womens Health Rep (New Rochelle). 2021 Nov 2;2(1):507–15. doi: 10.1089/whr.2021.0046 (PMC8617587; doi:10.1089/whr.2021.0046)
Supplement: Supplemental data [file Suppl_TableS2.docx]

**Supplemental Table 2. Definitions of additional variables used as predictors in model**

| **Variable** | **Definition** |
| --- | --- |
| Multiple gestation | ICD-9: 651.x-xx  ICD-10: O30.x-xxx |
| Obesity | ICD-9: 278.0x, 649.1x, V85.3, V85.4  ICD-10: E66.x-xx, 099.21x, Z68.3x, Z68.4x |
| Parity | Documented in the prenatal care record |
