# Supplementary material for: Development and Validation of an Obstetric Comorbidity Risk Score for Clinical Use
Source: Womens Health Rep (New Rochelle). 2021 Nov 2;2(1):507–15. doi: 10.1089/whr.2021.0046 (PMC8617587; doi:10.1089/whr.2021.0046)
Supplement: Supplemental data [file Suppl_TableS3.docx]

**Supplemental Table 3.** **Predictors in final Obstetric Comorbidity Score**

| **Predictor** ^a^ | **Development** ^b^  **N = 227,405** | **Validation** ^b^  **N= 41,683** | **Beta Coefficient** |
| --- | --- | --- | --- |
| **CCS 195:** Other complications of birth | 129335 (56.9) | 25139 (60.3) | 0.20 |
| **CCS 181:** Other complications of pregnancy | 125426 (55.2) | 28682 (68.8) | 0.05 |
| **Parity** |  |  |  |
| 0 | 101015 (44.4) | 18587 (44.6) | 0.91 |
| 1-4 | 124624 (54.8) | 22740 (54.6) | Reference |
| ≥5 | 1766 (0.8) | 356 (0.9) | 0.17 |
| **Maternal age, years** |  |  |  |
| ≤19 | 5581 (2.5) | 510 (1.2) | 0.24 |
| 20-24 | 22856 (10.1) | 3749 (9.0) | 0.20 |
| 25-29 | 52650 (23.2) | 9569 (23.0) | Reference |
| 30-34 | 81972 (36.0) | 15148 (36.3) | 0.05 |
| 35-39 | 50465 (22.2) | 10168 (24.4) | 0.21 |
| 40-44 | 12820 (5.6) | 2366 (5.7) | 0.44 |
| ≥45 | 1061 (0.5) | 173 (0.4) | 0.80 |
| **CCS 182:** Hemorrhage during pregnancy; abruptio placenta; placenta previa | 41836 (18.4) | 6872 (16.5) | 0.07 |
| **CCS 159:** Urinary tract infections | 34213 (15.0) | 6676 (16.0) | 0.07 |
| **CCS 189:** Previous cesarean section | 29899 (13.1) | 5233 (12.6) | 0.21 |
| **CCS 186:** Diabetes or abnormal glucose tolerance complicating pregnancy | 29376 (12.9) | 5855 (14.0) | 0.22 |
| **CCS 251:** Abdominal pain | 27048 (11.9) | 4781 (11.5) | 0.06 |
| **CCS 163:** Genitourinary symptoms and ill-defined conditions | 26942 (11.8) | 5321 (12.8) | 0.07 |
| **Obesity ^c^** | 22904 (10.1) | 7690 (18.4) | 0.28 |
| **CCS 174:** Female infertility | 17000 (7.5) | 3185 (7.6) | 0.07 |
| **CCS 257:** Other aftercare | 14335 (6.3) | 4618 (11.1) | 0.05 |
| **CCS 95:** Other nervous system disorders | 12174 (5.4) | 2202 (5.3) | 0.24 |
| **CCS 183:** Hypertension complicating pregnancy | 11612 (5.1) | 2205 (5.3) | 1.70 |
| **CCS 49:** Diabetes mellitus without complications | 11397 (5.0) | 2302 (5.5) | 0.21 |
| **CCS 117:** Other circulatory disease | 9680 (4.3) | 2673 (6.4) | 0.74 |
| **Multiple gestation** ^c^ | 7218 (3.2) | 3067 (7.4) | 0.66 |
| **CCS 92:** Otitis media and related conditions | 6773 (3.0) | 1128 (2.7) | 0.18 |
| **CCS 98:** Essential hypertension | 5578 (2.5) | 897 (2.2) | 0.85 |
| **CCS 62:** Coagulation and hemorrhagic disorders | 4642 (2.0) | 997 (2.4) | 0.37 |
| **CCS 149:** Biliary tract disease | 3537 (1.6) | 771 (1.8) | 0.29 |
| **CCS 53:** Disorders of lipid metabolism | 3298 (1.5) | 410 (1.0) | 0.18 |
| **CCS 151:** Other liver diseases | 2545 (1.1) | 477 (1.1) | 0.18 |
| **CCS 215:** Genitourinary congenital anomalies | 1346 (0.6) | 243 (0.6) | 0.25 |
| **CCS 50:** Diabetes mellitus with complications | 929 (0.4) | 135 (0.3) | 0.46 |
| **CCS 87:** Retinal detachments; defects; vascular occlusion; and retinopathy | 705 (0.3) | 113 (0.3) | 0.61 |
| **CCS 210**: Systemic lupus erythematosus and connective tissue disorders | 534 (0.2) | 102 (0.2) | 0.64 |
| **CCS 156:** Nephritis | 311 (0.1) | 33 (0.1) | 0.39 |
| **CCS 173:** Menopausal disorders | 291 (0.1) | 28 (0.1) | 0.61 |
| **CCS 158:** Chronic kidney disease | 234 (0.1) | 55 (0.1) | 1.02 |
| **CCS 654:** Developmental disorders | 189 (0.1) | 32 (0.1) | 0.87 |
| **CCS 54:** Gout and other crystal arthropathies | 53 (0.02) | 8 (0.02) | 0.99 |
| **CCS 99:** Hypertension with complications and secondary hypertension | 35 (0.02) | 5 (0.01) | 0.44 |
| **CCS 157:** Acute renal failure | 26 (0.01) | 7 (0.02) | 1.17 |
| **CCS 222:** Hemolytic jaundice and perinatal jaundice | 7 (0.00) | 1 (0.00) | 3.26 |
| **CCS 79:** Parkinson’s disease | 3 (0.00) | 0 (0.00) | 2.72 |
| **Intercept** | -- | -- | -4.60 |

CCS = Healthcare Cost and Utilization Project Clinical Classification Software

^a^ CCS descriptions have been abbreviated

^b^ Table values are n (column %); may not sum to 100% due to rounding (parity and maternal age)

^c^ Obesity and multiple gestation definitions can be found in Supplemental Table 2
