# Supplementary material for: Development and Validation of an Obstetric Comorbidity Risk Score for Clinical Use
Source: Womens Health Rep (New Rochelle). 2021 Nov 2;2(1):507–15. doi: 10.1089/whr.2021.0046 (PMC8617587; doi:10.1089/whr.2021.0046)
Supplement: Supplemental data [file Suppl_FigureS1.docx]

**Supplemental Figure 1. Performance metrics of Obstetrics Comorbidity Score across risk thresholds in development and validation cohorts**


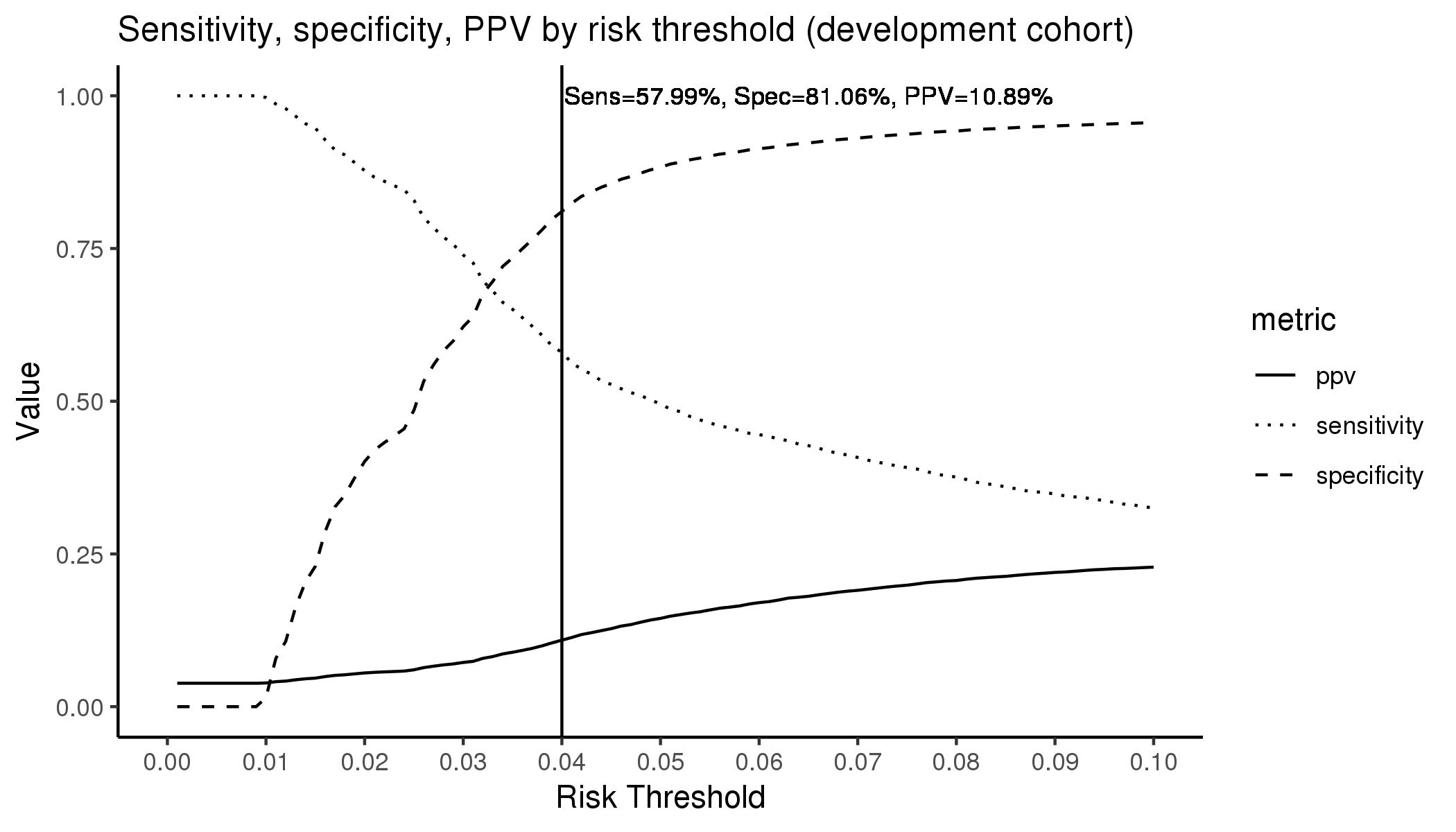


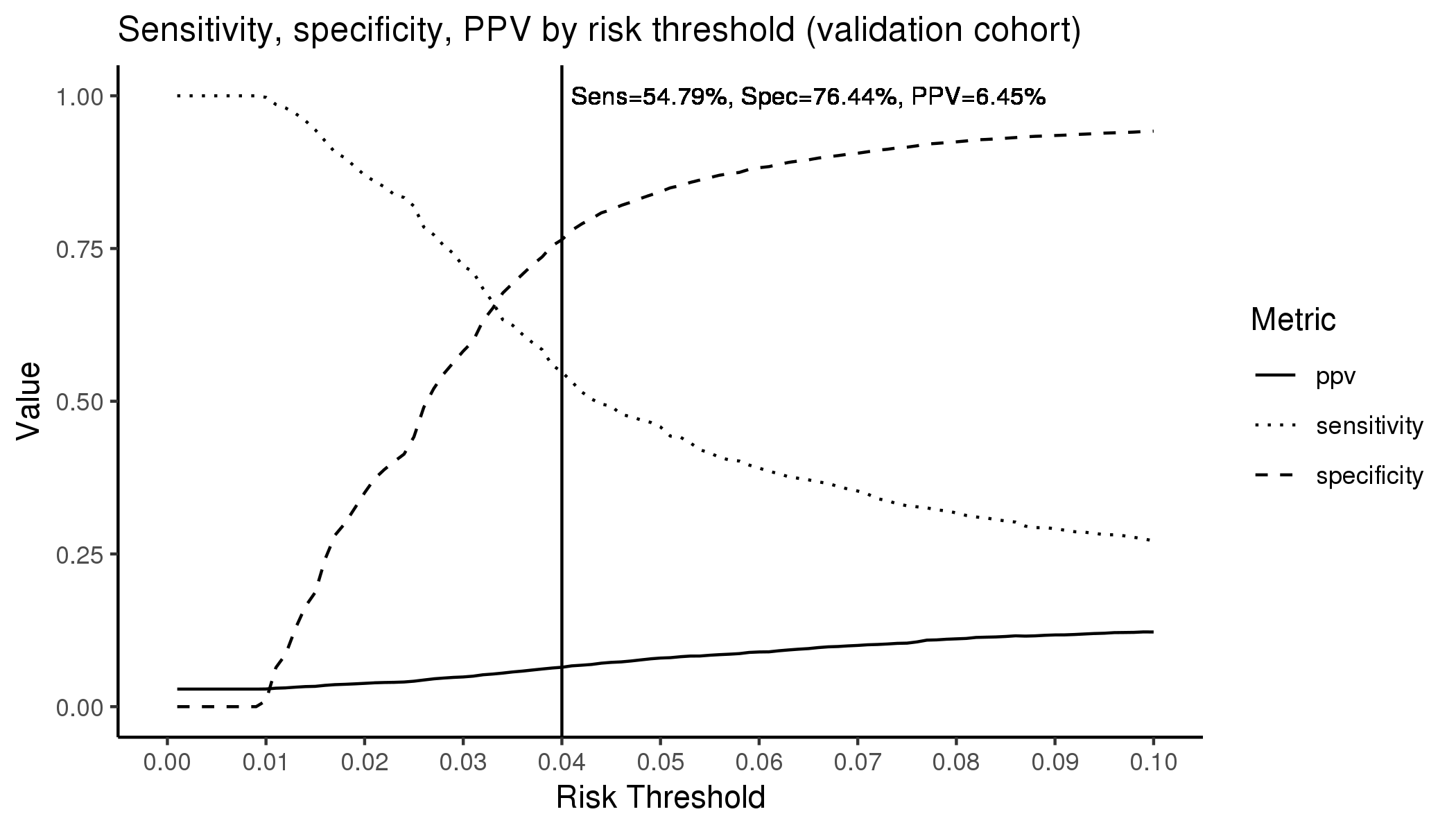


PPV = positive predictive value; Sens = sensitivity; Spec = specificity
