# Supplementary material for: Development and Validation of an Obstetric Comorbidity Risk Score for Clinical Use
Source: Womens Health Rep (New Rochelle). 2021 Nov 2;2(1):507–15. doi: 10.1089/whr.2021.0046 (PMC8617587; doi:10.1089/whr.2021.0046)
Supplement: Supplemental data [file Suppl_TableS4.docx]

**Supplemental Table 4. Comparison of percentage of outcomes captured by the Obstetric Comorbidity Score, simple risk factors, and Bateman Comorbidity Index (validation cohort)**

|  | **% pregnancies crossing threshold (n=41,683)** | **Outcomes identified (%)**  **(n=1,201)** | **Outcomes identified, excluding pre-eclampsia/eclampsia (%)**  **(n=500)** |
| --- | --- | --- | --- |
| **Admission for delivery hospitalization ^a^** | | | |
| OCS, 4% threshold | 24.5 | 54.8 | 40.8 |
| OCS, 5% threshold | 16.6 | 45.8 | 29.6 |
| OCS, 6% threshold | 12.6 | 39.1 | 21.6 |
| Simple risk factors ^b^ | 28.2 | 41.5 | 38.2 |
| Bateman Comorbidity Index, cutoff of score ≥ 2 | 24.5 | 44.5 | 41.2 |
| **18 weeks of pregnancy ^a^** | | | |
| OCS, 4% threshold | 14.4 | 34.1 | 26.4 |
| OCS, 5% threshold | 8.4 | 24.5 | 16.2 |
| OCS, 6% threshold | 5.8 | 19.4 | 12.2 |
| Simple risk factors ^b^ | 15.5 | 28.6 | 24.8 |

OCS = Obstetric Comorbidity Score

**^a^** Score values calculated using predictor data from healthcare encounters in the 12-month period prior to respective time point

^b^ Hypertension (CCS 98 or CCS 99) and/or diabetes (CCS 49 or CCS 50 or CCS 186) and/or multiple gestation (see Supplemental Table 2) and/or maternal age ≥ 40

**References**

1. Bateman BT, Mhyre JM, Hernandez-Diaz S, et al. Development of a comorbidity index for use in obstetric patients. *Obstet Gynecol.* 2013;122(5):957-965.
2. Escobar GJ, Gupta NR, Walsh EM, Soltesz L, Terry SM, Kipnis P. Automated early detection of obstetric complications: theoretic and methodologic considerations. *Am J Obstet Gynecol.* 2019;220(4):297-307.
